# The relationship between volleyball experience and mental rotation ability in university students: the mediating role of updating function

**DOI:** 10.3389/fpsyg.2026.1927911

**Published:** 2026-10-08

**Authors:** Donghai Huang, Dongyi Huang, Peixin Shi, Pengyun Zhang, Muhammad Nubli Abdul Wahab, Zhipu Wang, Heng Wang

**Affiliations:** 1 School of Physical Education, Ningxia Normal University, Guyuan, China; 2 College of Physical Education, Henan Normal University, Xinxiang, China; 3 Center for Human Science, University Malaysia Pahang Al-Sultan Abdullah, Kuantan, Malaysia; 4 College of Physical Education, Changwon National University, Changwon-si, Gyeongsangnam-do, Republic of Korea

**Keywords:** executive functions, mental rotation ability, university students, updating function, volleyball expertise

## Abstract

This study aims to explore the relationships among university students’ volleyball experience, executive functions, and mental rotation ability, and to elucidate the underlying mechanisms. Using a cross-sectional design, 60 university students were categorized into high (HVEG), medium (MVEG), and low (LVEG) volleyball experience groups, and their mental rotation ability and executive functions were assessed. Multiple linear regression was conducted, structural equation modeling was performed with Mplus 8.3, and mediation effects were tested using 5,000 bootstrap samples. Results showed that significant differences among the three groups in executive function and mental rotation ability (*p* < 0.05), with the HVEG showing significantly shorter reaction times than the LVEG. Among the sub-components of executive function, only updating function was significantly correlated with mental rotation ability (*p* < 0.01). Mediation analysis revealed that volleyball experience was significantly associated with mental rotation ability (direct effect: *β* = −0.492, 83.8% of the total effect), and the updating function played a partial mediating role in this relationship (indirect effect: *β* = −0.094, 16.2% of the total effect). In conclusion, greater volleyball experience was associated with better (faster) mental rotation performance in university students, with the updating function playing a partial mediating role in this association.

## 1 Introduction

Mental rotation, the ability to mentally rotate and transform spatial representations, is a fundamental spatial cognitive ability (Shepard and Metzler, 1971; Uttal et al., 2013). Mental rotation and related spatial abilities are closely involved in motor decision-making and the execution of open motor skills that demand continuous spatial judgments, such as ball sports (Geisen et al., 2024). Accumulating evidence indicates that sport expertise is associated with superior spatial performance: athletes outperform non-athletes on spatial tasks (Moreau et al., 2012; Voyer and Jansen, 2017), and young volleyball and tennis athletes show better visual–spatial capacity than non-athletes (Notarnicola et al., 2014). Moreover, spatial abilities are malleable and can be enhanced through training and experience (Uttal et al., 2013), suggesting that long-term sport participation may be an effective pathway for spatial cognitive development (Erickson et al., 2019). However, the cognitive mechanisms underlying the association between sport experience and mental rotation ability remain poorly understood. Executive functions, higher-order cognitive processes comprising inhibition, updating, and shifting (Friedman and Miyake, 2017; Miyake et al., 2000), represent a potential pathway, given their role in maintaining and revising spatial representations. Nevertheless, whether volleyball experience relates to mental rotation ability through executive functions has not been directly examined.

Volleyball, a typical open-skill sport, provides a suitable context for examining this relationship (Geisen et al., 2024). Volleyball is characterized by complex technical–tactical behaviors that demand rapid decision-making under time pressure (Maneiro et al., 2026). Playing volleyball requires players to continuously judge the ball’s flight trajectory and the spatial positions of teammates and opponents in real time (Rodrigues and De Sá Teixeira, 2024), and to continuously perform bodily spatial transformations and make motor decisions (Amara et al., 2024a). Compared to closed-skill sports such as weightlifting, long-term volleyball training is associated with optimized sensorimotor system characteristics and enhanced mental rotation ability (Voyer and Jansen, 2017). Meta-analytic evidence further shows that high-level volleyball athletes outperform both closed-skill athletes and lower-level volleyball players on mental rotation tasks (Feng et al., 2017). From a neurocognitive perspective, mental rotation depends on spatial processing networks centered on the parietal cortex, particularly the intraparietal sulcus (Zacks, 2008; Hiew et al., 2023). Because volleyball demands continuous spatial transformation and location judgments, long-term volleyball training may strengthen these parietal spatial processing networks through experience-dependent plasticity (Moreau et al., 2012), thereby facilitating mental rotation ability. Accordingly, this study proposes Hypothesis 1: greater volleyball experience is associated with better (faster) mental rotation performance in university students.

Embodied cognition theory posits that human cognition is rooted in bodily experiences, motor systems, and interactions with the environment (Wilson and Golonka, 2013). Within this framework, mental rotation is not merely a static manipulation of shapes but relies more heavily on bodily representations, motor simulation, and transformations of spatial reference frames. The updating function within executive functions may play a key role in this process (Ebert et al., 2024). The updating function refers to an individual’s ability to continuously update, replace, and dynamically process information in working memory, fundamentally involving the maintenance and reconstruction of spatial representations (D'Esposito, 2007; Buschman and Miller, 2023). Mental rotation tasks require individuals to continuously update spatial relationships, adjust bodily reference frames, and maintain dynamic spatial representations (Pardo-Vazquez and Fernandez-Rey, 2012). Previous studies have found that individuals with higher updating function typically exhibit faster reaction times in mental rotation tasks, indicating that updating function may be a crucial cognitive foundation for mental rotation ability (Zhao et al., 2026). Meanwhile, volleyball demands that players process complex information within extremely short time windows and dynamically adjust attention allocation and motor decisions according to environmental changes (Zhu et al., 2024). This process requires not only the rapid identification of relevant information but also the timely clearing out irrelevant information while maintaining effective working memory representations. Long-term volleyball experience is closely associated with higher executive function processing efficiency and information updating ability (Finkenzeller et al., 2021; Vona et al., 2024). Notably, the updating function and mental rotation ability share some overlap in neural mechanisms, both involving coordinated processing within the frontoparietal network (Wang et al., 2026; Yao et al., 2024). Therefore, the updating function may serve as an important cognitive mediator linking volleyball experience and mental rotation ability. Accordingly, this study proposes Hypothesis 2: the updating function statistically mediates the association between volleyball experience and mental rotation ability.

In summary, based on embodied cognition theory, the present study constructs a mediation model (Figure 1) that links university students’ volleyball experience to their mental rotation ability. In this model, the updating function is hypothesized to mediate the association between volleyball experience and mental rotation ability. The aim is to explore the cognitive mechanisms underlying the association between open-skill sport experience and spatial cognitive ability.

**Figure 1:**
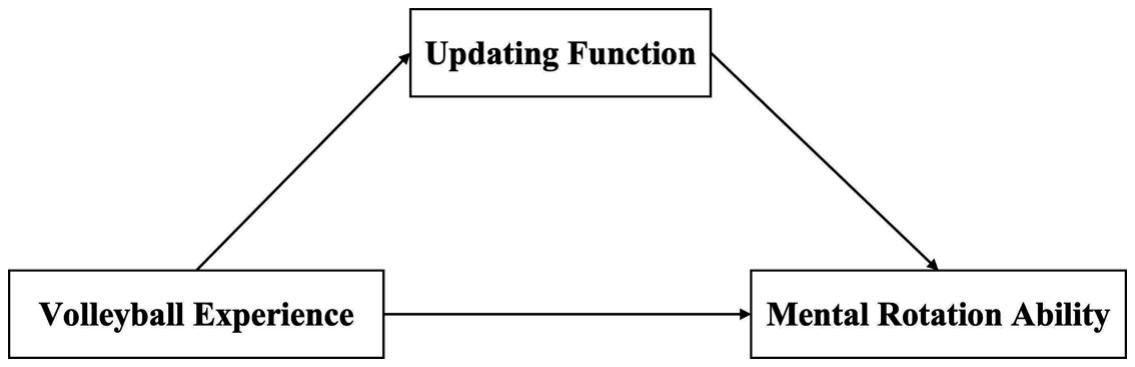
Hypothetical model.

## 2 Method

### 2.1 Participants

This study recruited 60 university students from Henan Normal University as participants, who were divided into three groups based on their volleyball experience levels: The High Volleyball Expertise Group (HVEG), the Medium Volleyball Expertise Group (MVEG), and the Low Volleyball Expertise Group (LVEG) (Table 1). Specifically, the HVEG included individuals who had participated in provincial-level or higher competitions and held a national Level 1 or above athlete certificate; the MVEG comprised those with 6 months to 2 years of professional training but had not reached Level 1; and the LVEG consisted of individuals without athletic classification and with only 1 to 6 months of volleyball training experience. Although there were some age differences among the groups, all participants were young adults with a narrow age range (18–24 years). All participants had normal or corrected-to-normal vision, no history of neurological disorders, psychiatric illnesses, or major physical diseases, and had not participated in other psychological or sports experiments. All participants were in good physical condition and capable of completing the cognitive experimental task. To minimize the influence of external factors on cognitive test results, all participants were instructed to avoid alcohol consumption, staying up late, and strenuous exercise for 24 h prior to the experiment. All statistical analyses reported in this study were based on this final sample of 60 participants.

**Table 1:** Basic information of participants.

Group	*n*	Average age	Years of training
High Volleyball Expertise Group	20 (Males 12, Females 8)	20.85 ± 1.59	7.75 ± 2.04
Moderate Volleyball Expertise Group	20 (Males 10, Females 10)	22.25 ± 0.96	2.00 ± 0.57
Low Volleyball Expertise Group	20 (Males 10, Females 10)	20.50 ± 0.76	0.55 ± 0.10

### 2.2 Measurement

Executive Functions. Inhibition, updating, and shifting were assessed using the Flanker task, the 2-back task, and the More-odd shifting task, respectively, administered with E-Prime 3.0 software on a laptop. All participants received standardized instructions and completed practice trials before the formal test, and only those who reached at least 80% accuracy in the practice phase proceeded to the formal phase. The Flanker task measured inhibition as the difference between reaction times (RTs) in incongruent and congruent conditions (Eriksen and Eriksen, 1974); the 2-back task measured updating as the mean RT of correct responses (Smith and Jonides, 1997); and the More-odd shifting task measured shifting as the RT difference between switching and non-switching conditions (Salthouse et al., 2003). Detailed task parameters and instructions are provided in the Supplementary Figures S1–S8.

Mental rotation ability. Mental rotation ability was measured using the R-letter mental rotation task, which assesses object-based mental rotation ability (Snow, 1990). The task required participants to quickly determine whether the stimulus on the right was a regular or mirror image while maintaining accuracy: if the right figure could be aligned with the left letter R by clockwise rotation, it was identified as a normal image (press the F key); otherwise, it was identified as a mirror image (press the L key). The stimuli included both normal and mirror images, each presented at six rotation angles: 0°, 60°, 120°, 180°, 240°, and 300°. The formal experiment consisted of 72 trials, preceded by 12 practice trials. The mean reaction time of correct trials was used as the performance metric; shorter reaction times indicate higher mental rotation processing efficiency.

### 2.3 Statistical methods

All data were organized and analyzed using SPSS 29.0. In the present study, volleyball experience was represented by the three expertise groups, which were ordinally coded as 0 (low), 1 (medium), and 2 (high), with higher scores indicating greater volleyball experience. The structural equation mediation model was considered the primary analysis; the ANOVA, correlation, and regression analyses served as preliminary and supplementary examinations. (a) One-way ANOVA and repeated-measures ANOVA were conducted to test for differences among the three groups, and LSD tests were used for *post hoc* pairwise comparisons following significant results. (b) Pearson correlation analysis was used to examine the relationships between volleyball experience, executive function subcomponents, and mental rotation ability. (c) Multiple linear regression models relating volleyball experience, executive function subcomponents, and mental rotation ability were constructed using R (the lm function). (d) Structural equation modeling was conducted using Mplus 8.3, and mediation effects were tested via bootstrap resampling with 5,000 iterations. (e) The statistical power to detect the indirect effect was evaluated using a Monte Carlo sensitivity analysis based on the observed effect sizes (Supplementary Table S5). All statistical tests were two-tailed, with a significance level set at *p* < 0.05. Because all participants were young adults with a narrow age range, age was not included as a covariate in the primary analyses.

## 3 Results

### 3.1 Comparison of executive functions among the three groups

Results revealed significant differences among the three groups in inhibition (HVEG: 91.41 ± 24.01 ms; MVEG: 98.08 ± 35.63 ms; LVEG: 120.31 ± 39.56 ms), updating (HVEG: 800.73 ± 75.75 ms; MVEG: 858.18 ± 79.75 ms; LVEG: 907.50 ± 107.41 ms), and shifting (HVEG: 237.08 ± 89.21 ms; MVEG: 308.51 ± 82.67 ms; LVEG: 368.80 ± 134.39 ms) (*p* < 0.05), with the HVEG exhibiting shorter reaction times than the other two groups across all tasks (Table 2). Further pairwise comparisons (Supplementary Table S2) indicated that the HVEG and LVEG differed significantly in inhibition, updating, and shifting functions (*p* < 0.01 or *p* < 0.001). The HVEG and MVEG showed significant differences in updating and shifting (*p* < 0.05), but not in inhibition. The MVEG and LVEG only differed significantly in inhibition (*p* < 0.05), with no significant differences in updating and shifting.

**Table 2:** Comparison of executive function subcomponents across different volleyball expertise levels.

Executive functions (ms)	HVEG	MVEG	LVEG	*F*	*p*	$\eta_{p}^{2}$
Inhibition	91.41 ± 24.01	98.08 ± 35.63	120.31 ± 39.56	4.026	0.023*	0.12
Updating	800.73 ± 75.75	858.18 ± 79.75	907.50 ± 107.41	7.249	0.002*	0.20
Shifting	237.08 ± 89.21	308.51 ± 82.67	368.80 ± 134.39	7.939	0.001***	0.22

HVEG = High Volleyball Expertise Group; MVEG = Moderate Volleyball Expertise Group; LVEG = Low Volleyball Expertise Group. *p < 0.05, **p < 0.01, ***p < 0.001.

### 3.2 Comparison of mental rotation ability among university students with the three groups

Results revealed significant differences in mental rotation ability among the three groups (HVEG: 873.19 ± 140.03 ms; MVEG: 974.24 ± 167.44 ms; LVEG: 1105.07 ± 203.85 ms), with the HVEG showing the shortest reaction times, followed by the MVEG and then the LVEG (Supplementary Table S1). Repeated measures ANOVA showed that the main effect of group was significant [*F*(2, 57) = 9.092, *p* < 0.01, $\eta_{p}^{2}$ =0.24], the main effect of rotation angle was significant [*F*(5, 285) = 124.588, *p* < 0.001, $\;\eta_{p}^{2}$ =0.69], and the interaction between group and rotation angle was not significant [*F*(10, 285) = 1.421, *p* > 0.05, $\eta_{p}^{2}$ =0.05]. Pairwise comparisons showed that the HVEG differed significantly from the LVEG (*p* < 0.01), the MVEG differed significantly from the LVEG (*p* < 0.05), but the HVEG did not differ significantly from the MVEG (*p* > 0.05) (Supplementary Table S2), suggesting that greater disparities in experience level between groups were associated with larger differences in mental rotation ability.

Analysis of the mean reaction times across rotation angles (Figure 2) revealed that the HVEG exhibited shorter reaction times than both the MVEG and LVEG at all rotation angles. Across all three groups, reaction times exhibited an inverted U-shaped pattern as rotation angle increased: the longest reaction times occurred at 180°, followed by 120° and 240°, then 60° and 300°, with the shortest reaction times at 0°. The differences in reaction times among the three groups were largest at 180° and smallest at 0°, indicating that the trend of reaction time changes across angles was consistent across groups, all displaying an inverted U-shaped distribution.

**Figure 2:**
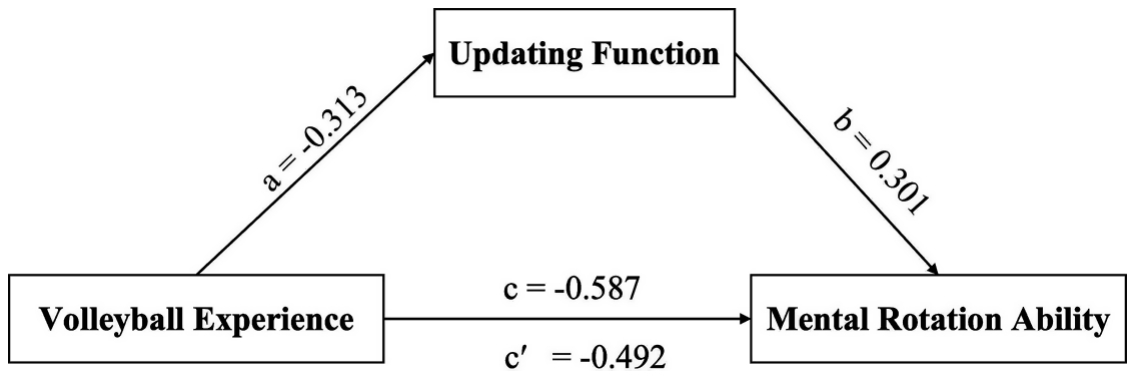
Mean reaction times across rotation angles for mental rotation ability among the three groups of participants.

### 3.3 Correlation analysis between executive functions and mental rotation ability

Results (Table 3) indicated that executive functions and mental rotation ability were correlated across the three groups. Specifically, the updating function was significantly positively correlated with mental rotation ability, whereas inhibition and shifting did not show consistent correlations.

**Table 3:** Correlation analysis between executive functions and mental rotation ability.

Variable	1	2	3	4	5	6	7	8	9	10
1. Inhibition	–									
2. Updating	0.046	–								
3. Shifting	−0.239	0.079	–							
4. Mental Rotation	0.072	0.641**	0.123	–						
5. 0°	0.032	0.472*	0.090	0.769**	–					
6. 60°	0.158	0.602**	0.075	0.865**	0.783**	–				
7. 120°	0.047	0.631**	0.092	0.891**	0.649**	0.701**	–			
8. 180°	0.078	0.624**	−0.060	0.743**	0.311	0.513*	0.651**	–		
9. 240°	0.035	0.423	0.198	0.915**	0.705**	0.712**	0.738**	0.619**	–	
10. 300°	0.011	0.387	0.262	0.767**	0.579**	0.740**	0.552*	0.355	0.761**	–

*p < 0.05, **p < 0.01, ***p < 0.001.

### 3.4 Predictive contribution of executive functions to mental rotation ability

Results of the multiple linear regression analysis (Table 4) indicated that, among the three predictors included, the updating function made a significant contribution to mental rotation ability. This effect was evident across the high-experience group (*p* < 0.01), the moderate-experience group (*p* < 0.01), and the low-experience group (*p* < 0.05). Further analysis examined which rotation angles best explained updating-function performance within each group (Table 4). For the MVEG, reaction time at 120° was a significant predictor of updating-function performance (*p* < 0.01); for the LVEG, reaction time at 0° was a significant predictor (*p* < 0.05). For the HVEG, the best-fitting model included reaction times at 120° and 180°, but neither predictor reached significance (*p* = 0.109 and *p* = 0.122, respectively). Overall, the updating function emerged as an important predictor of mental rotation ability among university students across the three experience levels.

**Table 4:** Model selection results for each group.

Dependent variable	Adj. *R^2^*	AIC	BIC	Best model
HVEG Mental Rotation Ability	0.4	−22.11147	−19.12427	Updating function (*p* = 0.00171)
MVEG Mental Rotation Ability	0.27	−16.08948	−12.10655	Updating function (*p* = 0.0092)
LVEG Mental Rotation Ability	0.25	−14.23582	−11.24862	Updating function (*p* = 0.0141)
HVEG Updating Function	0.45	−43.53918	−39.55625	120° (*p* = 0.109) + 180° (*p* = 0.122)
MVEG Updating Function	0.55	−48.02238	−44.03945	120° (*p* = 0.00107)
LVEG Updating Function	0.27	−30.24873	−27.26154	0° (*p* = 0.0138)

AIC = Akaike information criterion; BIC = Bayesian information criterion.

### 3.5 Mediation effect analysis

The mediation model was a saturated model (df = 0); model fit indices are reported in Supplementary Table S3. The results of the mediation analysis (Table 5 and Figure 3) indicated that the direct effect of volleyball experience on mental rotation ability was −0.492 (95% CI: [−0.641, −0.313]), which did not include 0, indicating a significant direct effect (83.8% of the total effect). The indirect effect via the updating function was −0.094 (95% CI: [−0.201, −0.017]), which also did not include 0, indicating a significant indirect effect (16.2% of the total effect). Further analysis indicated that the model demonstrated good explanatory power for mental rotation ability (*R*^2^ = 0.426; *R*^2^ significance tests are reported in Supplementary Table S4), suggesting that volleyball experience and updating function together explain mental rotation ability. Therefore, volleyball experience showed a significant direct association with mental rotation ability as well as an indirect association through the updating function.

**Table 5:** Mediation effect analysis of volleyball experience, updating function, and mental rotation ability.

Effect type	Path	*β*	Boot *SE*	*p*	95%CI		Ratio to total effect
					LLCI	ULCI	
Direct Effects	Volleyball Expertise → Mental Rotation Ability (c′)	−0.492	0.085	<0.001	−0.641	−0.313	83.8
Indirect Effect (Mediation)	Volleyball Expertise → Updating Function→ Mental Rotation Ability	−0.094	0.048	0.047	−0.201	−0.017	16.2
	Volleyball Expertise → Updating Function (a)	−0.313	0.107	0.004	−0.503	−0.081	
	Updating Function → Mental Rotation Ability (b)	0.301	0.096	0.002	0.121	0.497	
Total Effect	Volleyball Expertise → Mental Rotation Ability	−0.587	0.072	<0.001	−0.712	−0.430	100

**Figure 3:**
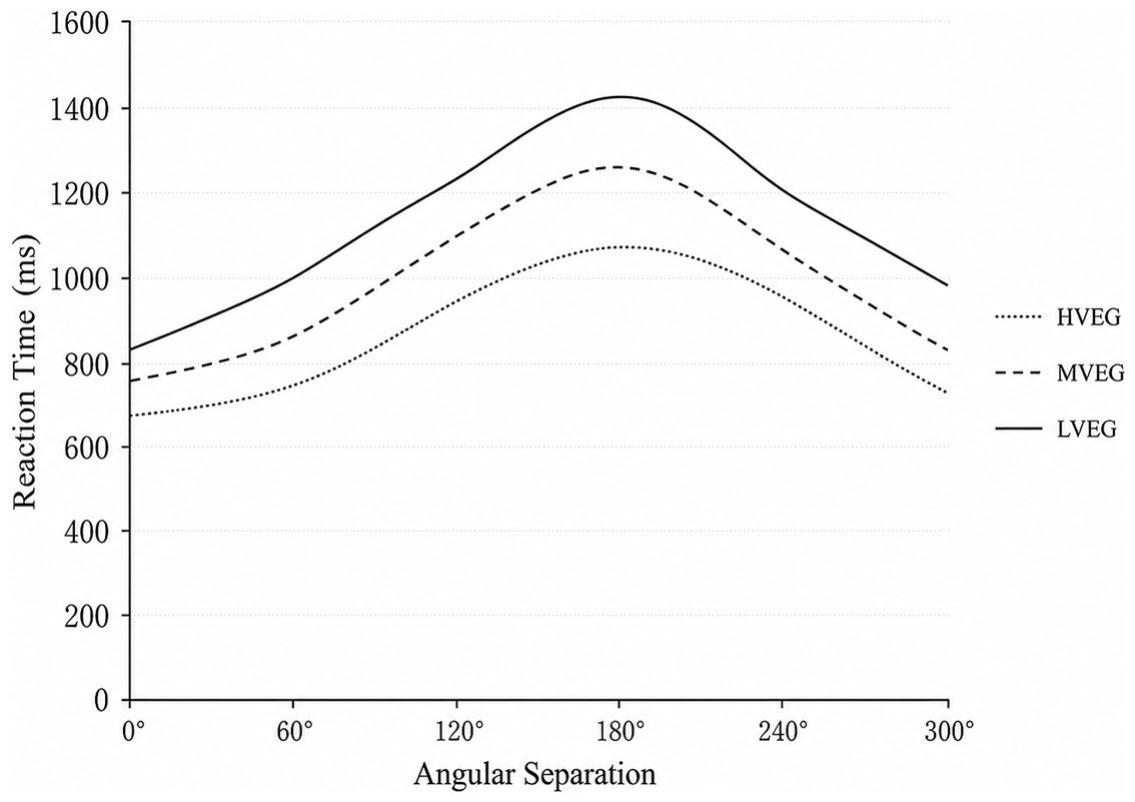
Mediation model of the updating function.

## 4 Discussion

This study constructed and tested a mediation model with the updating function as the mediating variable, examining the relationships among volleyball experience, executive functions, and mental rotation ability. The results first revealed significant differences among the three experience groups in both executive functions and mental rotation ability, with the HVEG consistently outperforming the LVEG. Furthermore, volleyball experience was significantly associated with mental rotation ability and this association was partially mediated by the updating function. These findings shed light on the potential cognitive mechanisms underlying the association between open motor skill experience and spatial cognitive ability, providing a theoretical reference for cultivating spatial cognitive ability in university students.

### 4.1 The relationship between volleyball experience and mental rotation ability

Greater volleyball experience was associated with better mental rotation performance, consistent with Hypothesis 1. This finding is consistent with previous research on the relationship between motor experience and spatial cognition. Meta-analyses and empirical studies have consistently shown that open motor skill experience (in soccer, gymnastics, and ball sports) is positively and stably correlated with performance on spatial tasks such as mental rotation (Jansen and Lehmann, 2013; Voyer and Jansen, 2017). The present study extends this finding to the context of volleyball, providing direct evidence for the volleyball player population.

Embodied cognition theory posits that mental rotation is intimately linked to bodily experience and the motor system (Barsalou, 2008; Doganci et al., 2023). Consistently, Wexler et al. (1998) found that motor processes are directly involved in mental rotation processing. Volleyball players are required to constantly process dynamic spatial information, judge the ball’s flight trajectory, anticipate the positions of teammates and opponents, and quickly execute bodily spatial transformations. This “perception-action” coupling experience may facilitate the development of spatial representation ability (Raab, 2020; Huesmann and Loffing, 2024). The present study further found that the mental rotation advantage of more experienced players was more pronounced at the 180° condition, that is, under higher cognitive load, suggesting that motor experience is related to a greater behavioral advantage in high cognitive load situations. This finding may offer new insights into the cognitive conditions under which sport-related advantages in mental rotation emerge, although this requires further empirical verification.

### 4.2 The mediating role of the updating function

The present study found that the updating function played a partial mediating role between volleyball experience and mental rotation ability, thereby supporting Hypothesis 2. This finding is consistent with existing theoretical frameworks in which updating ability often serves as a mediator in “input–output” relationships, such as the pathway through which sleep quality affects academic performance via updating function (Haoyu et al., 2023). Embodied cognition theory suggests that the mental rotation advantage observed in athletes after long-term training results from the interaction between bodily experience and cognitive processing, and that greater motor experience is associated with better mental rotation performance (Jansen et al., 2012).

The present study extends previous research in the following ways. First, although some studies have examined the mental rotation ability of athletes in sports such as soccer and gymnastics (Klotzbier and Schott, 2024) and have identified features of visuospatial cognitive abilities in volleyball players, to our knowledge, no previous study has directly tested the mediating mechanisms underlying these effects. The present study is the first to our knowledge to test this mediation pathway (“motor experience → updating function → mental rotation ability”) in volleyball players. Second, the present study clarified the specific roles of executive function subcomponents, suggesting that, among the three subcomponents, only the updating function served as a mediator. This finding is consistent with previous research suggesting that different subcomponents of executive functions play distinct roles across various tasks (Lee and Engle, 2026). It is also worth noting that, for the HVEG, the best-fitting model included reaction times at 120° and 180°, but neither reached significance (*p* = 0.109 and *p* = 0.122, respectively). This null result may be attributable to the small subgroup size (*n* = 20) and the consequently limited statistical power for detecting within-group associations, rather than to an absence of the underlying relationship.

In this study, the statistically significant indirect effect via the updating function accounted for 16.2% of the total association, indicating that the updating function explained only a relatively small portion of the relationship between volleyball experience and mental rotation ability, consistent with the partial (rather than full) mediation observed. This proportion should therefore be interpreted with caution: it indicates that the updating function is one of possibly several pathways, but it should not be taken as evidence that the updating function is the sole or primary cognitive mechanism, because mediation proportions derived from cross-sectional data are sensitive to measurement error and unmeasured confounding, and because, with only 20 participants per group, the estimate may be unstable across samples. Accordingly, the mediation effect should be regarded as an initial finding that awaits replication in larger, independent samples. Other mediating pathways are likely to be involved, and sport specificity deserves particular attention in this regard (Bartseva et al., 2024). Previous studies have shown that athletes from different sports exhibit distinct performance characteristics in mental rotation tasks (Weigelt and Memmert, 2021; Ben Mehrez et al., 2026). For instance, volleyball and badminton players exhibit group differences when performing mental rotation tasks under dynamic balance conditions, and high-level volleyball players demonstrate specific advantages in visuospatial cognition (Amara et al., 2024b). Therefore, sport specificity may be an important factor influencing the magnitude of the mediating effect, and future research could further examine the moderating role of sport type on this mediating pathway, ideally with longitudinal or experimental designs.

Our findings are consistent with embodied cognition theory, which posits that cognitive processes are deeply rooted in the body’s interactions with its environment rather than being isolated symbolic operations. The observed association between greater volleyball experience and better mental rotation performance, along with the mediating role of the updating function, provides cross-sectional correlational support for this theory in the domain of volleyball. Specifically, the complex spatial tracking and motor planning required in volleyball are not merely physical executions; they are closely related to higher-order spatial representations and executive control processes (Feng et al., 2024; Gu et al., 2019). However, given the cross-sectional design of the current study, future longitudinal or experimental research is needed to further verify the causal direction of these embodied cognitive pathways.

## 5 Limitations and future directions

Several limitations of this study should be acknowledged. First, this study relied solely on behavioral measures (reaction time) to assess executive functions and mental rotation ability, without employing neuroimaging techniques such as electroencephalography (EEG), functional near-infrared spectroscopy (fNIRS), or functional magnetic resonance imaging to verify the underlying neural mechanisms; the interpretation of the findings is therefore primarily based on theoretical inferences from existing literature. In addition, reaction time served as the primary index in the 2-back task; to reduce the risk of speed-accuracy trade-off effects, participants were required to reach an accuracy of at least 80% in the practice phase before the formal test, and reaction times were computed exclusively from correct trials. Second, a major limitation of this study is its cross-sectional design, which precludes definitive causal inferences regarding the relationships among volleyball experience, updating function, and mental rotation ability. Although we proposed a directional hypothesis based on embodied cognition theory, the observed associations cannot rule out reverse causality. It is equally plausible that individuals with inherently higher spatial cognitive and updating abilities are more likely to excel in volleyball, thereby accumulating more sports experience. Furthermore, unmeasured third variables, such as general cognitive ability, educational background, motivation, participation in other sports, or genetic predispositions, may confound these associations. Consequently, the findings should be interpreted as correlational evidence supporting the embodied cognition framework rather than direct proof of causation. Future research should employ longitudinal designs or randomized controlled trials (novice volleyball interventions) to establish definitive causal pathways. Third, the sample size was relatively small (*n* = 60; 20 per group). A Monte Carlo sensitivity analysis based on the observed effect sizes indicated that the power to detect the indirect effect was approximately 0.56, and that approximately 90 participants would be needed to reach the conventional 0.80 level (Supplementary Table S5). The mediation finding should therefore be regarded as preliminary and requires confirmation in larger, independent samples. Fourth, participants were recruited from a single university, which limits the generalizability of the findings. Fifth, gender distribution and gender effects were not analyzed, and although the MVEG was slightly older on average than the other two groups, all participants were young adults (18–24 years) with a narrow age range and age was therefore not entered as a covariate; the potential influence of these demographic differences was not fully ruled out. Future studies could recruit larger and more diverse samples with gender-balanced groups, apply mediation-specific power analyses, and combine behavioral measures with neuroimaging techniques to further examine the neural mechanisms through which sport experience influences spatial cognition.

## 6 Conclusion

The present study found that volleyball experience was associated with mental rotation ability in university students, with the updating function playing a partial mediating role in this relationship. This finding deepens the understanding of the potential cognitive mechanisms linking open-skill sport experience with spatial cognitive ability via the updating function. It also offers preliminary empirical support for embodied cognition theory in the domain of volleyball and offers theoretical implications for physical–cognitive integrated training approaches.

## Data Availability

The original contributions presented in the study are included in the article/Supplementary material, further inquiries can be directed to the corresponding author.
