# Supplementary material for: The relationship between volleyball experience and mental rotation ability in university students: the mediating role of updating function
Source: Front Psychol. 2026 Oct 8;17:1927911. doi: 10.3389/fpsyg.2026.1927911 (PMC13689573; doi:10.3389/fpsyg.2026.1927911)
Supplement: Supplementary file 1 [file Data_Sheet_1.PDF]

Supplementary Material

Detailed Descriptions of the Executive Function Tasks

All three tasks were administered with E-Prime 3.0 software on a laptop. Before the formal test, all participants received standardized instructions and completed practice trials; only participants who reached at least 80% accuracy in the practice phase proceeded to the formal phase. Reaction times were recorded in milliseconds, and the performance indices were based on correct responses.

Flanker task (inhibition). The task comprised a practice phase (12 trials) and a formal phase (48 trials). Each trial began with a fixation cross presented for 500 ms, followed by a row of arrows presented for 1,000 ms, with an inter-trial interval of 2,000 ms. Participants were required to respond to the direction of the central arrow while ignoring the flanking arrows (congruent vs. incongruent conditions). The inhibition index was computed as the difference between the mean RT in the incongruent condition and the mean RT in the congruent condition, with smaller differences indicating better inhibitory control. The instructions are presented in Supplementary Figure S1 and Figure S2.

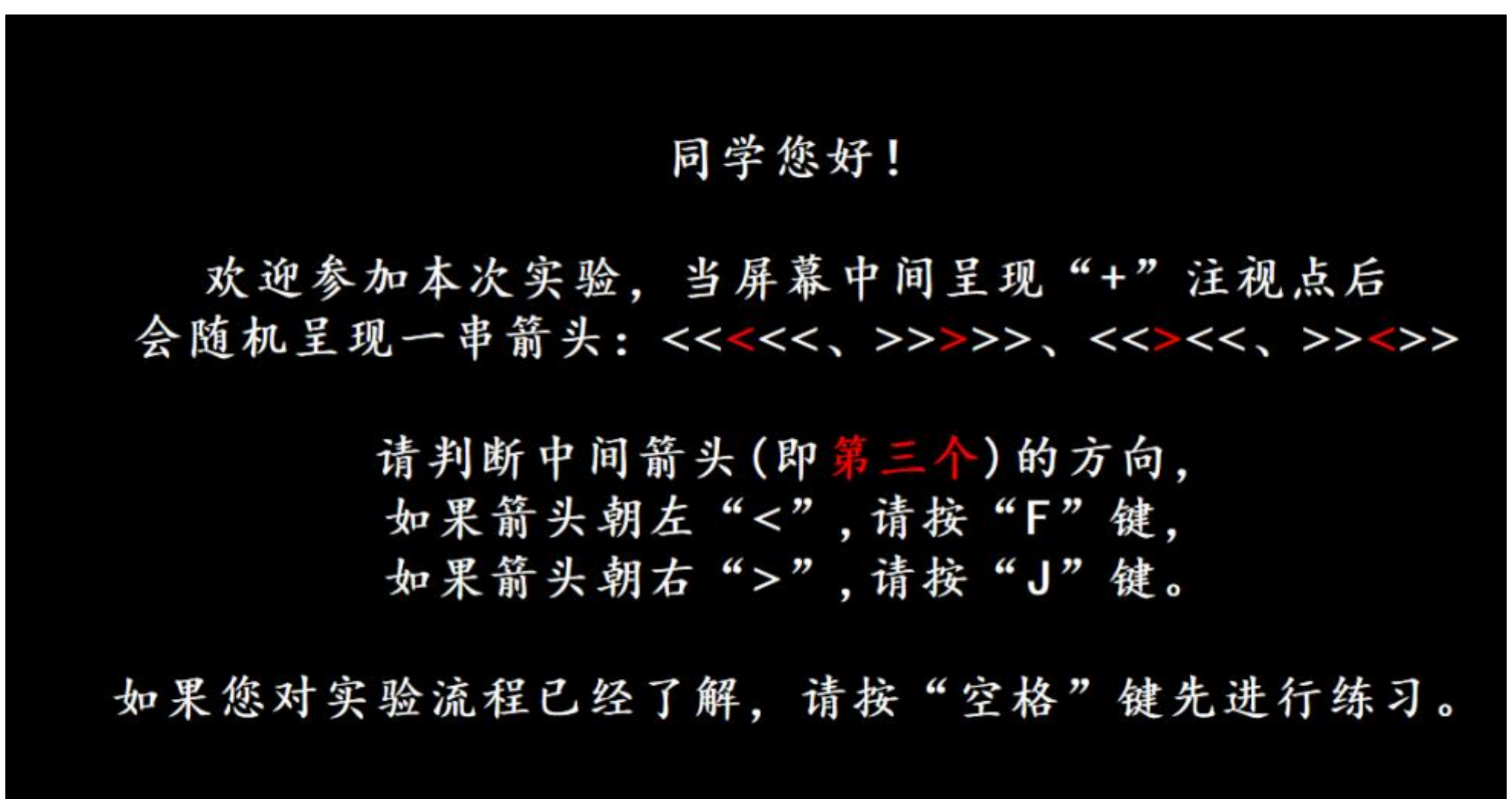

Figure S1 Flanker task prompt (Chinese)

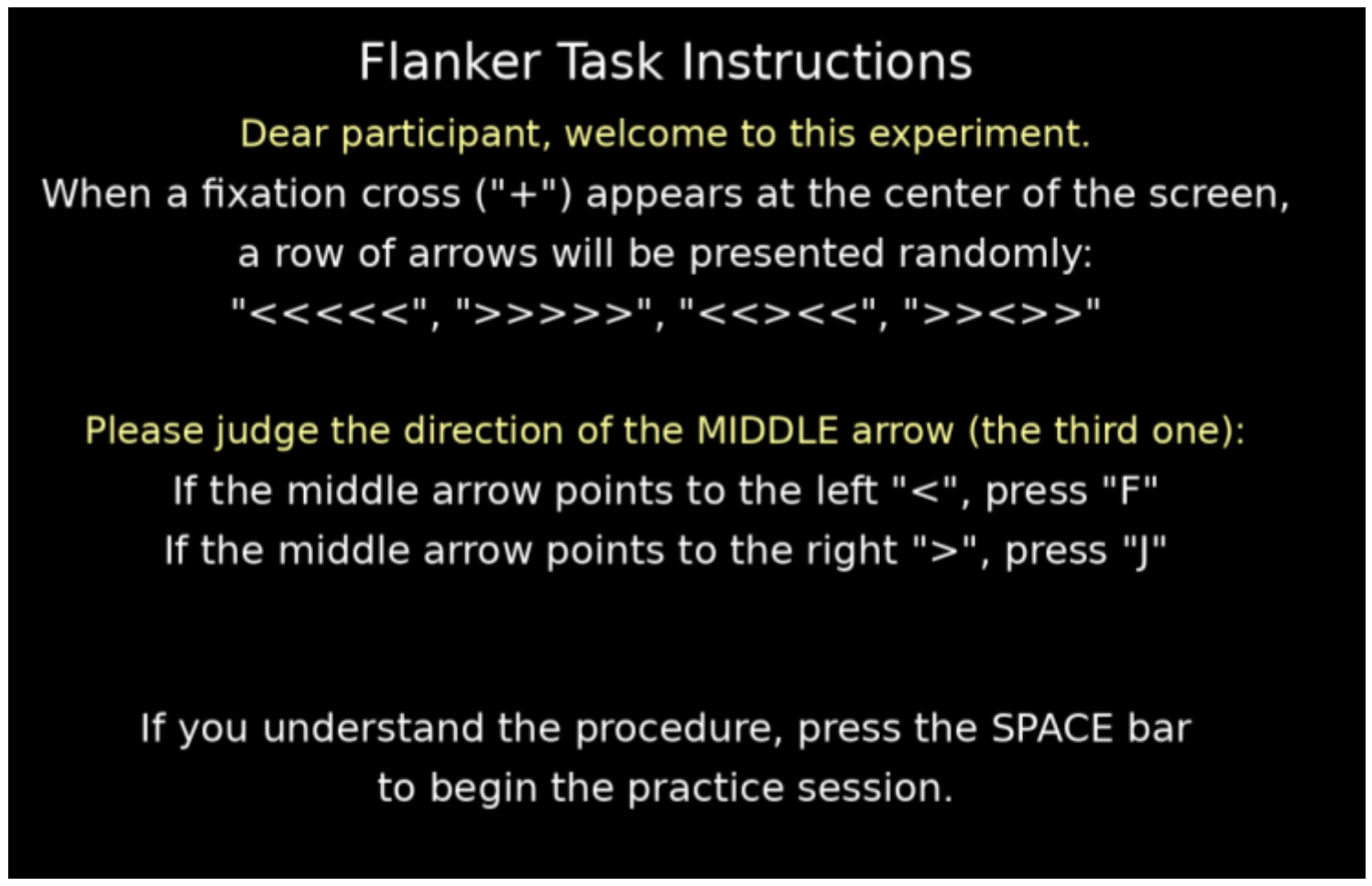

Figure S2 Instructions for the flanker task (English translation)

2-back task (updating). The updating function was assessed using a 2-back task (Smith et al.,1997). Five uppercase letters (Q, W, E, R, T) were presented sequentially at the center of the screen; each letter was displayed for 2,000 ms with an inter-stimulus interval of 3,000 ms. Participants were asked to judge whether the current letter matched the one presented two trials earlier, pressing "F" for a match and "J" for a mismatch. The task consisted of a practice phase (12 trials) and a formal phase (25 trials); only participants who reached an accuracy of at least 80% in the practice phase proceeded to the formal test. Both reaction time and accuracy were recorded, and the mean reaction time of correct responses served as the primary index, with shorter RTs indicating better updating function. The instructions are presented in Supplementary Figure S3 and Figure S4.

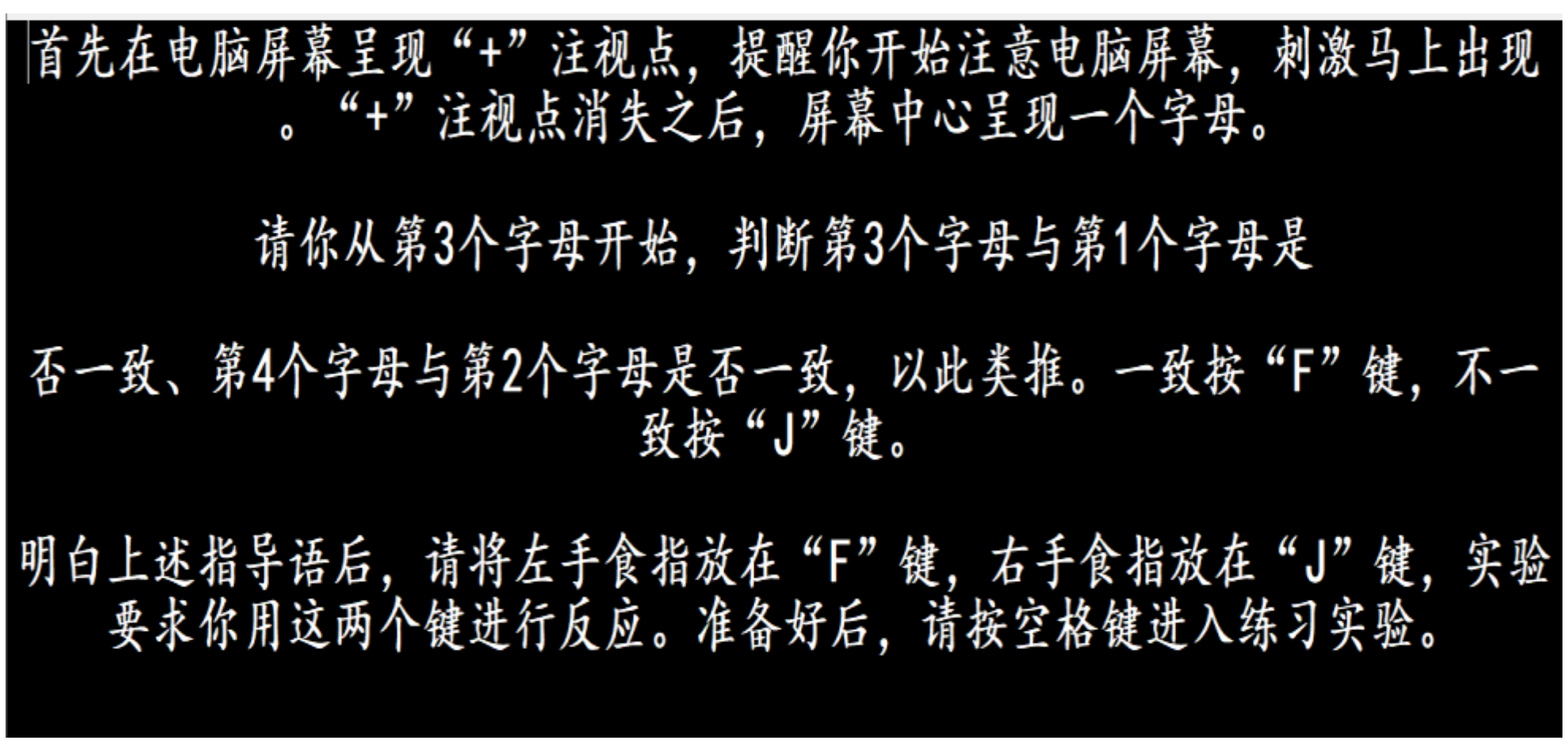

Figure S3 2-back task prompt (Chinese)

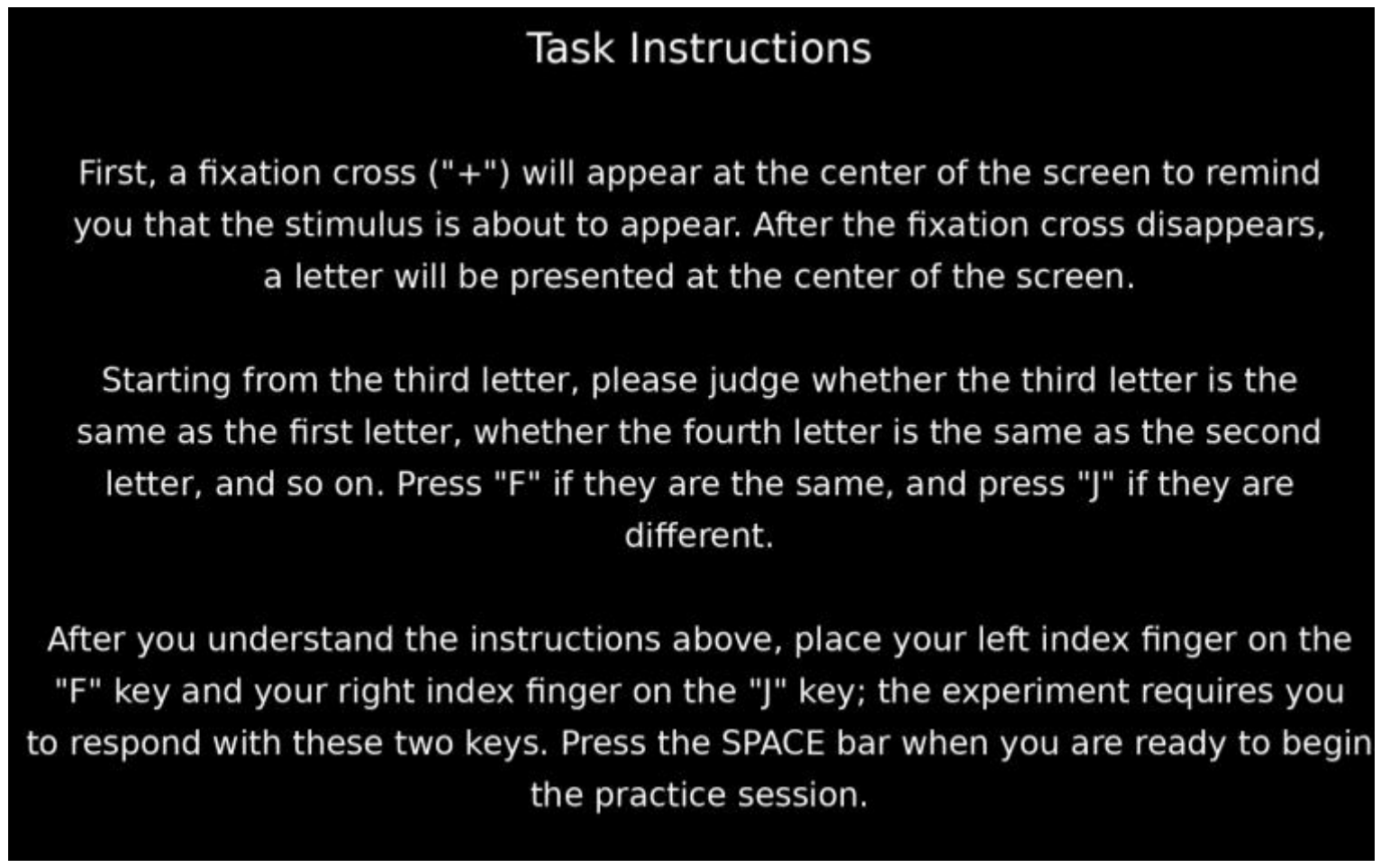

Figure S4 Instructions for the 2-back task (English translation)

More-odd shifting task (shifting). Digits from 1 to 9 were presented randomly on the screen, with each stimulus displayed for 2,000 ms and an inter-stimulus interval of 3,000 ms. The task included three conditions: (a) magnitude judgment—white digits, participants judged whether the digit was smaller or larger than 5 (pressing "F" for <5 and "J" for >5); (b) odd/even judgment—green digits, participants judged whether the digit was odd or even (pressing "F" for odd and "J" for even); and (c) mixed condition—the judgment rule switched according to the color of the digit. The shifting index was computed as the difference between the mean RT of switching trials and the mean RT of non-switching trials, with smaller differences indicating better shifting ability. The instructions are presented in Supplementary Figure S5, Figure S6, Figure S7, Figure S8.

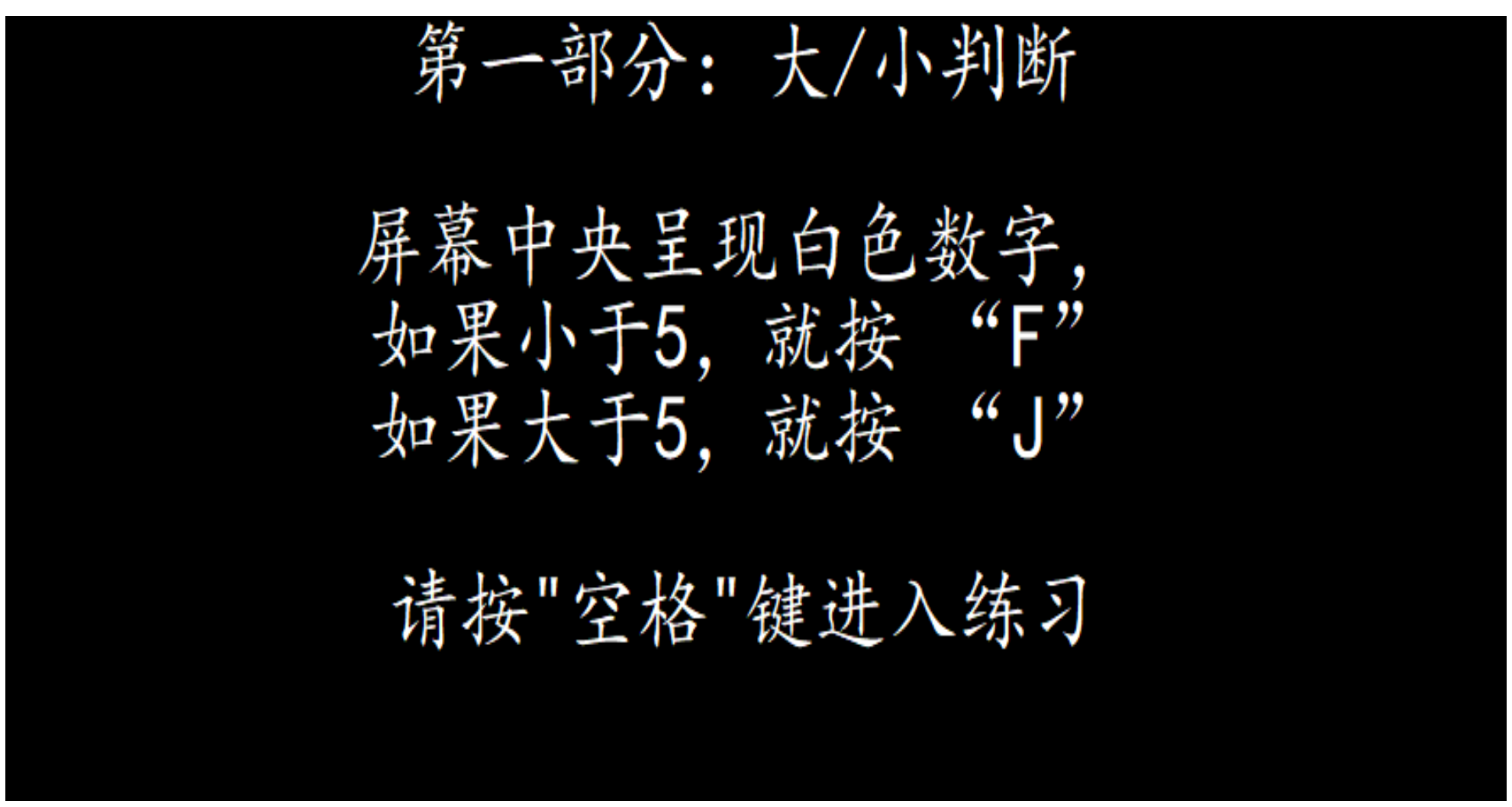

Figure S5 More-odd shifting（magnitude judgment）prompt (Chinese)

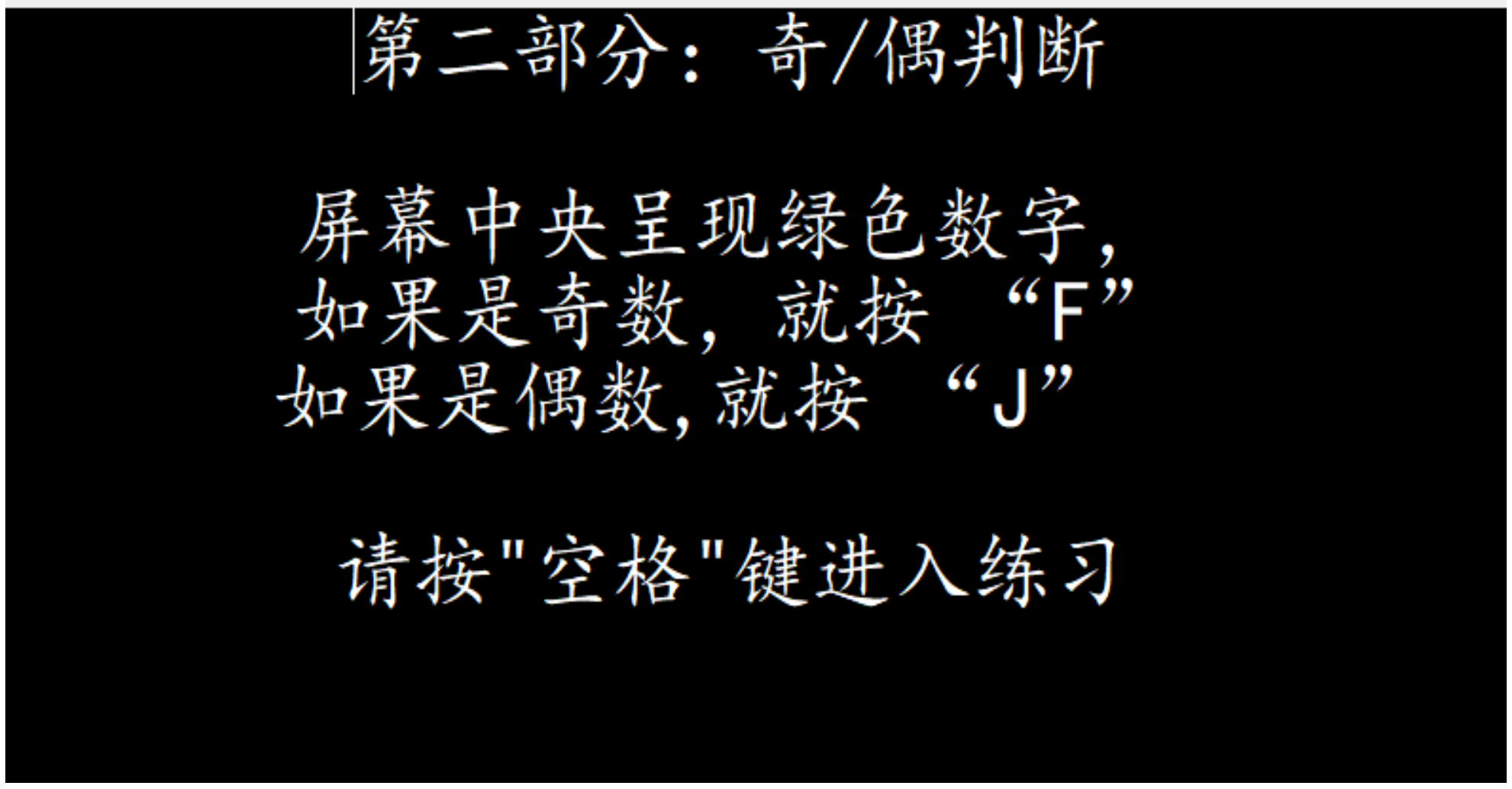

Figure S6 More-odd shifting（odd/even judgment）prompt (Chinese)

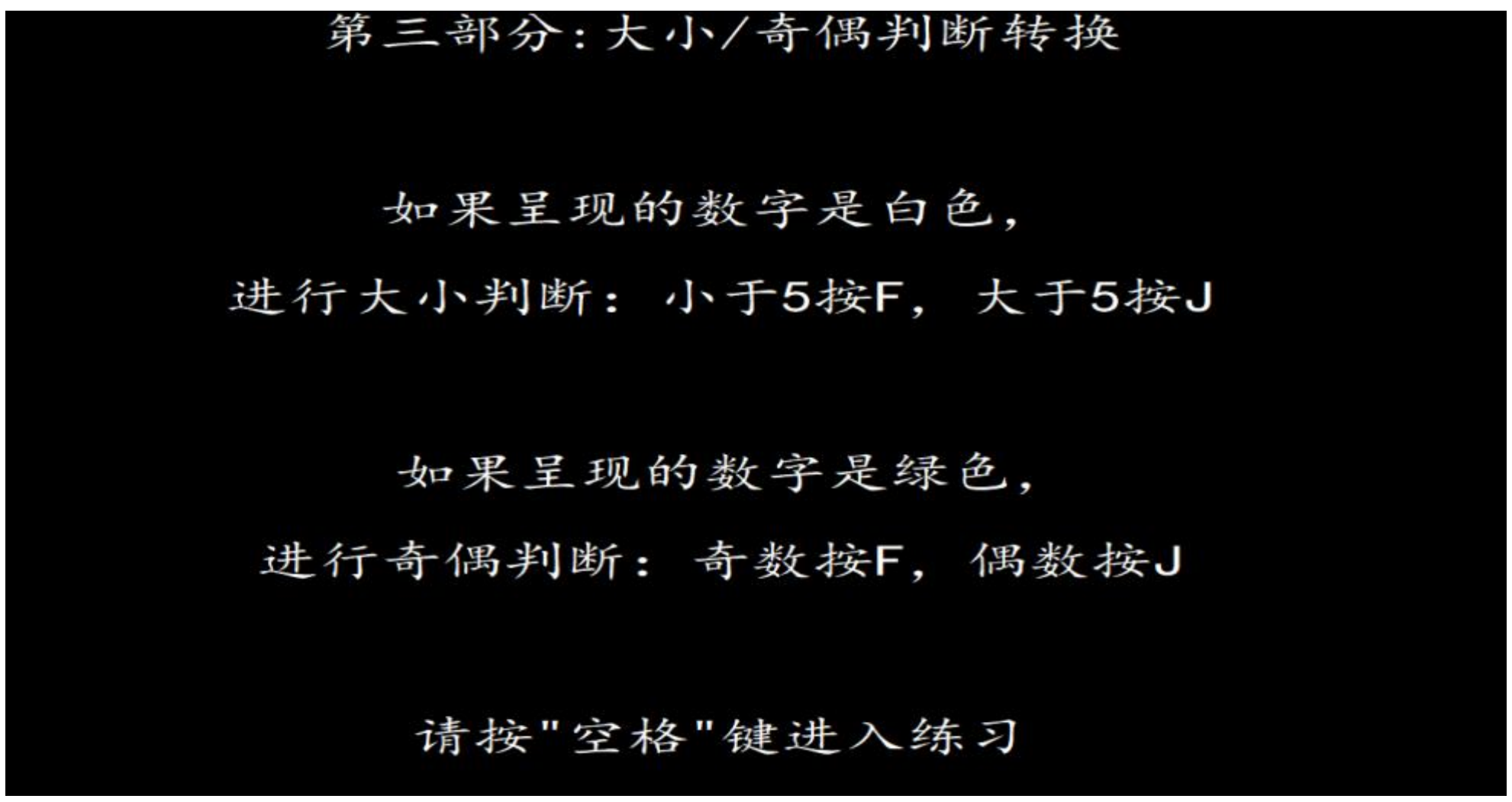

Figure S7 More-odd shifting（magnitude judgment, odd/even judgment）prompt (Chinese)

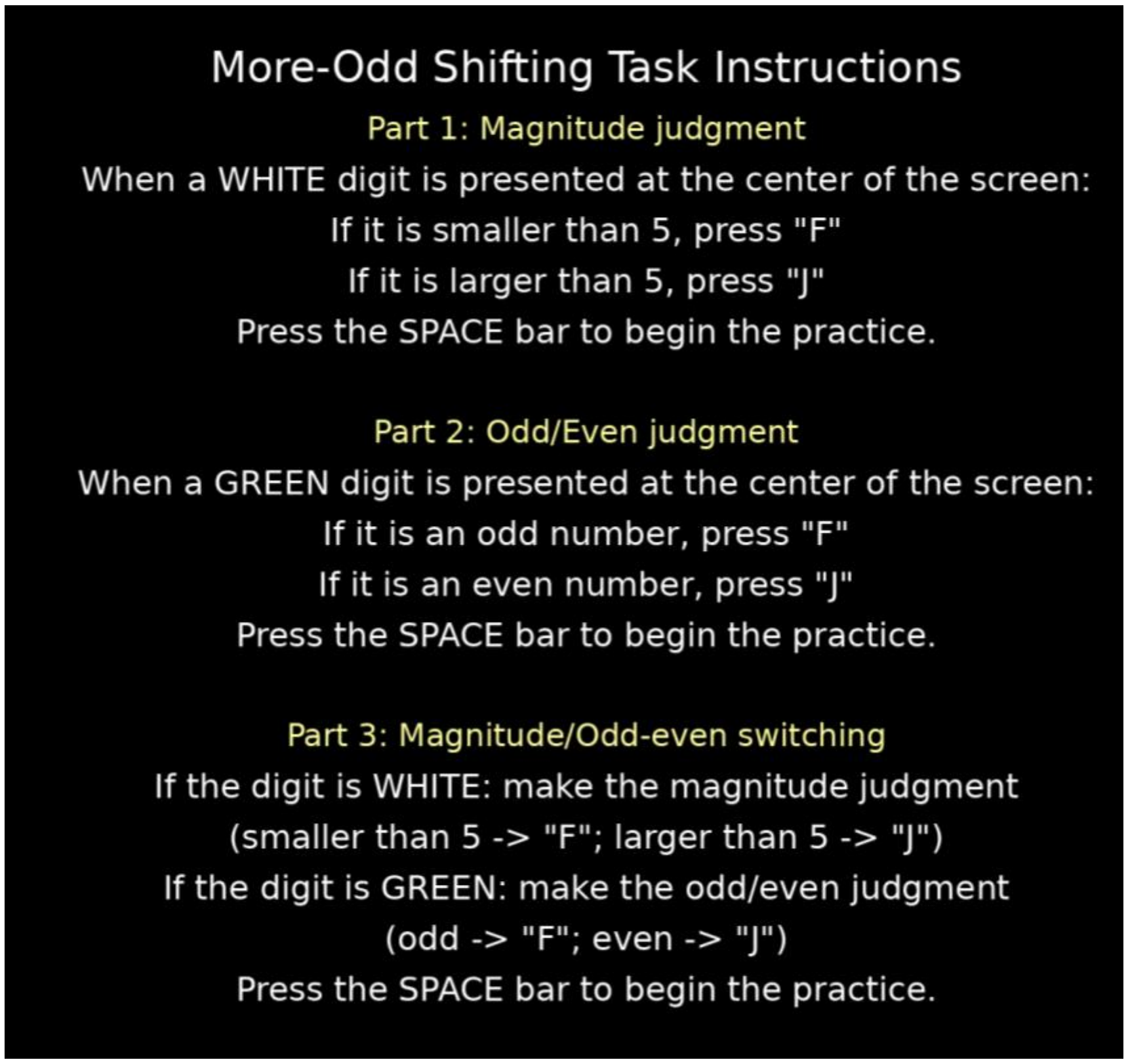

Figure S8 Instructions for the More-odd shifting task (English translation; Parts 1–3: magnitude judgment, odd/even judgment, and switching) (English translation)

Table S1 Reaction Times of Mental Rotation Tasks Across Different Volleyball Expertise Levels

	HVEG	MVEG	LVEG
Mental Rotation（ms）	873.19±140.03	974.24±167.44	1105.07±203.85
0°（ms）	678.97±108.36	756.81±112.48	832.73±125.42
60°（ms）	771.39±140.40	872.00±148.26	991.93±213.63
120°（ms）	978.44±231.25	1079.71±190.27	1217.43±300.71
180°（ms）	1074.92±195.77	1261.04±270.07	1425.14±250.60
240°（ms）	980.87±208.26	1047.34±278.11	1187.45±291.62
300°（ms）	754.58±121.75	828.90±164.80	976.04±234.20

Table S2 Multiple Comparisons of Executive Function Subcomponents and Mental Rotation Across Different Volleyball Experience Levels (LSD)

	（I）Group	（J）Group	Mean Difference（I-J）	SE	p
Inhibition	HVEG	MVEG	-6.66150	10.66467	0.535
		LVEG	-28.89700	10.66467	0.009**
	MVEG	LVEG	-22.23550	10.66467	0.042*
Updating	HVEG	MVEG	-57.44850	28.06953	0.045*
		LVEG	-106.77250	28.06953	<0.001***
	MVEG	LVEG	-49.32400	28.06953	0.084
Shifting	HVEG	MVEG	-71.42750	33.09442	0.035*
		LVEG	-131.71800	33.09442	<0.001***
	MVEG	LVEG	-60.29050	33.09442	0.074
Mental Rotation	HVEG	MVEG	-101.04450	54.52917	0.069
		LVEG	-231.87750	54.52917	<0.001***
	MVEG	LVEG	-130.83300	54.52917	0.020*

Note. * $p < 0.05$, ** $p < 0.01$, *** $p < 0.001$.

Table S3 Fit indices of the saturated mediation model

Index	χ^2	CFI	TLI	RMSEA	SRMR
Value	0.000 （0）	1.000	1.000	0.000 [0.000, 0.000]	0.000

Note. The model was saturated (df = 0), so conventional fit indices are at their perfect values and do not discriminate; model acceptability was evaluated through path coefficients and bootstrap 95% confidence intervals.

Table S4 R^2 and significance tests of the mediation model

Variable	R^2	SE	Est./S.E.	p
Updating function	0.098	0.065	1.504	0.133
Mental rotation	0.426	0.076	5.578	< 0.001

Table S5 Monte Carlo sensitivity analysis of statistical power for detecting the indirect effect

Sample size	Estimated power
60 (present study)	0.56
70	0.65
80	0.76
90	0.81
100	0.88
110	0.90
150	0.98

Note. Power was estimated by Monte Carlo simulation (1,200–1,500 replications) based on the unstandardized path coefficients and residual variances observed in the present study ($a = -39.483$; $b = 0.598$). For each replication, the indirect effect ($a \times b$) was tested using percentile bootstrap confidence intervals with 5,000 resamples, consistent with the main analysis. Power refers specifically to the detection of the indirect effect.
